# Risk Factors and Indices of Osteomyelitis of the Jaw in Osteoporosis Patients: Results from a Hospital-Based Cohort Study in Japan

**DOI:** 10.1371/journal.pone.0079376

**Published:** 2013-11-01

**Authors:** Toru Yamazaki, Masashi Yamori, Shiro Tanaka, Keiichi Yamamoto, Eriko Sumi, Megumi Nishimoto-Sano, Keita Asai, Katsu Takahashi, Takeo Nakayama, Kazuhisa Bessho

**Affiliations:** 1 Department of Oral and Maxillofacial Surgery, Graduate School of Medicine, Kyoto University, Kyoto, Japan; 2 Department of Pharmacoepidemiology, School of Public Health, Graduate School of Medicine, Kyoto University, Kyoto, Japan; 3 Department of Preventive Medicine and Epidemiologic Informatics, Research and Development Initiative Center, National Cerebral and Cardiovascular Center, Osaka, Japan; 4 Department of Clinical Innovative Medicine, Translational Research Center, Kyoto University, Kyoto, Japan; 5 Department of Health Informatics, School of Public Health, Graduate School of Medicine, Kyoto University, Kyoto, Japan; University Hospital of the Albert-Ludwigs-University Freiburg, Germany

## Abstract

**Background:**

Several studies have reported osteomyelitis of the jaw (OMJ) as a side effect of bisphosphonates (BPs), and the risk of oral BPs has been recently clarified. However, other systemic risk factors of OMJ remain unclear. Importantly, the possibility of risk classification based on the clinical characteristics of patients has not been explored. Here, we clarified risk factors of OMJ and evaluate the predictive accuracy of risk indices in osteoporosis patients.

**Methods:**

We performed sub-analysis using a database developed for a retrospective cohort study in patients taking medications for osteoporosis at Kyoto University Hospital. Risk indices for OMJ were constructed using logistic regression analysis, and odds ratios (OR) for OMJ cases and 95% confidence intervals (CI) were estimated. Potential risk factors included in the statistical analysis were age; sex; diabetes; use of oral BPs, corticosteroids, cancer chemotherapy, antirheumatic drugs, and biologic agents; and their interactions. Risk indices were calculated by the sum of potential risk factors of an individual patient multiplied by the regression coefficients. The discriminatory power of the risk indices was assessed by receiver operating characteristic (ROC) analysis.

**Results:**

In analysis of all patients, oral BPs (OR: 4.98, 95% CIs: 1.94-12.75), age (OR: 1.28, 95% CI: 1.06-1.60) and sex-chemotherapy interaction (OR: 11.70, 95% CI: 1.46-93.64) were significant risk factors of OMJ. Areas under the ROC curves of these risk indices provided moderate sensitivity or specificity regardless of group (0.683 to 0.718).

**Conclusions:**

Our data suggest that oral BP use, age, and sex-chemotherapy are predictors of OMJ in osteoporosis patients. The risk indices are moderately high, and allow the prediction of OMJ incidence.

## Introduction

Oral bisphosphonates (BPs) are useful in the treatment of various bone metabolic diseases, such as postmenopausal osteoporosis or Paget disease [1], but their use is associated with the occurrence of osteonecrosis of the jaw (ONJ), also known as osteomyelitis of the jaw (OMJ), as an adverse effect. This condition is considered refractory and causes a decrease in the quality of life in patients. Although the risk of OMJ with oral BPs was initially unclear, possibly as a result of its low incidence, recent large studies have demonstrated the incidence rate of OMJ in oral BPs users or the relative risk of oral BPs in osteoporosis patients after adjustment for potential risk factors [2-10].

Several academic associations have published position papers and guidelines which suggest that practitioners should beware of the incidence of OMJ in BPs users, particularly patients with other potential systematic risk factors for OMJ, such as diabetes, corticosteroid use and cancer chemotherapy [11-14]. Most previous studies did not clarify in detail the association between systemic factors and the incidence of OMJ [2-6,8,9], however, and the risk factors have yet to be identified and remain speculative [15]. Only one study has shown a correlation, and this was a single correlation only and was made without adjustment for oral BP use [7]. Furthermore, the prediction of OMJ on the basis of risk factors has not been established. Thus, effective decision making based on risk assessment of OMJ in osteoporosis patients is hampered by a lack of etiological information.

Here, we investigated potential systemic risk factors for OMJ and evaluated the predictive accuracy of risk indices for OMJ using data from our hospital-based cohort study in patients with osteoporosis.

## Methods

### Study design and cohort

We performed sub-analysis using a database previously constructed for a retrospective cohort study conducted at Kyoto University Hospital from February 2011 to July 2012 [10]. Subjects were diagnosed with osteoporosis as specified by the 10th edition of the International Classification of Diseases (ICD-10) code at Kyoto University Hospital between November 2000 and October 2010 (Appendix S1) and prescribed osteoporosis medications approved in Japan (Appendix S2). Among these patients, analysis was limited to those aged 20 years or older who had been treated with osteoporosis medications. This criterion was based on previous findings that age at first onset of BP-related ONJ was approximately 20 years [16-18].

We then excluded patients who had the presence of primary or metastatic tumors, a history of trauma or radiation therapy in the maxillofacial region, or treatment with intravenous BPs.

### Data extraction

Hospital data were extracted from the electronic medical records (EMR) using an EMR retrieval system [19]. This system retrieves electronic data for both outpatients and inpatients at Kyoto University Hospital, including demographic data, diagnosis and ICD-10 code, medications and injections, laboratory tests, radiological or pathological studies, etc. We used this system to search for patients who were diagnosed with osteoporosis, OMJ, or other diseases as specified by ICD-10 code, then reviewed patients with diseases possibly related to OMJ (Appendix S3).

### Outcome measurements

Although several academic societies have stated that the hallmark of BP-related ONJ is exposed necrotic bone in the maxillofacial region that has persisted for more than 8 weeks [12,13,20], we consider it difficult to distinguish ONJ from OMJ and accordingly propose grouping cases of OMJ together with ONJ. There are two reasons for this: first, radiographic ﬁndings in infected jawbone in patients treated with BPs are similar to those in BP-induced ONJ even if necrotic bone cannot be clinically visualized [21-23]; and second, the presence of osteonecrosis is a common histopathologic ﬁnding in both BP-induced ONJ and OMJ [24]. These finding suggest that the presence of bone exposure in the oral cavity is not always caused by avascular necrosis of the jaw. Several studies or reviews have also regarded ONJ as the same as OMJ [10,25-27].

OMJ was independently reconfirmed by two trained oral and maxillofacial surgeons using proposed criteria based on findings obtained from panoramic X-ray, technetium bone scan, computed tomography, histological picture or surgery, either alone or in combination. Inter-observer agreement was moderate (kappa value = 0.64 to 0.81). Detailed information on patients and methods is reported in our previous work [10,19].

### Potential risk factors

The following risk factors were included into the statistical analysis: age, sex, diabetes; use of oral BPs, corticosteroids, cancer chemotherapy, antirheumatic drugs, and biologic agents; and the interactions of potential risk factors. Diabetes was diagnosed if the patient had received a diagnosis of diabetes, and had either received any treatment with hypoglycemic medication (hypoglycemic agent and/or insulin) or had an HbA1c ≥6.5% [28]. Steroid use was defined as the receipt of any treatment with corticosteroids, and chemotherapy, antirheumatic drugs and biologic agents use as the receipt of any treatment with cancer chemotherapy, antirheumatic drugs and biologic agents.

### Statistical analysis

Patient characteristics were summarized using descriptive statistics (range and percentages). The risk indices for OMJ were constructed using logistic regression analysis with OMJ as the dependent variable. Odds ratios (OR) for OMJ cases and 95% confidence intervals (CI) were estimated in all patients taking any osteoporosis medication and in the subset of oral BP users. Risk indices were calculated by the sum of the potential risk factors of an individual patient multiplied by the regression coefficients. The discriminatory power of the risk indices was assessed by receiver operating characteristic (ROC) analysis. All *P* values were two-sided at the significance level of 5%. All statistical analyses were performed using SAS Version 9.2 (SAS Institute, Cary, NC, USA).

### Ethics Statement

The protocol for this study was approved by the Ethics Committee of Kyoto University Graduate School and Faculty of Medicine and the study was conducted according to the Declaration of Helsinki. We did not obtain written informed consent because this is a retrospective study. The Ethics Committee follows the ethical guidelines for epidemiological research of the Japanese Ministry of Health, Labour and Welfare, and accordingly does not require written informed consent for these studies.

## Results

**Figure 1:**
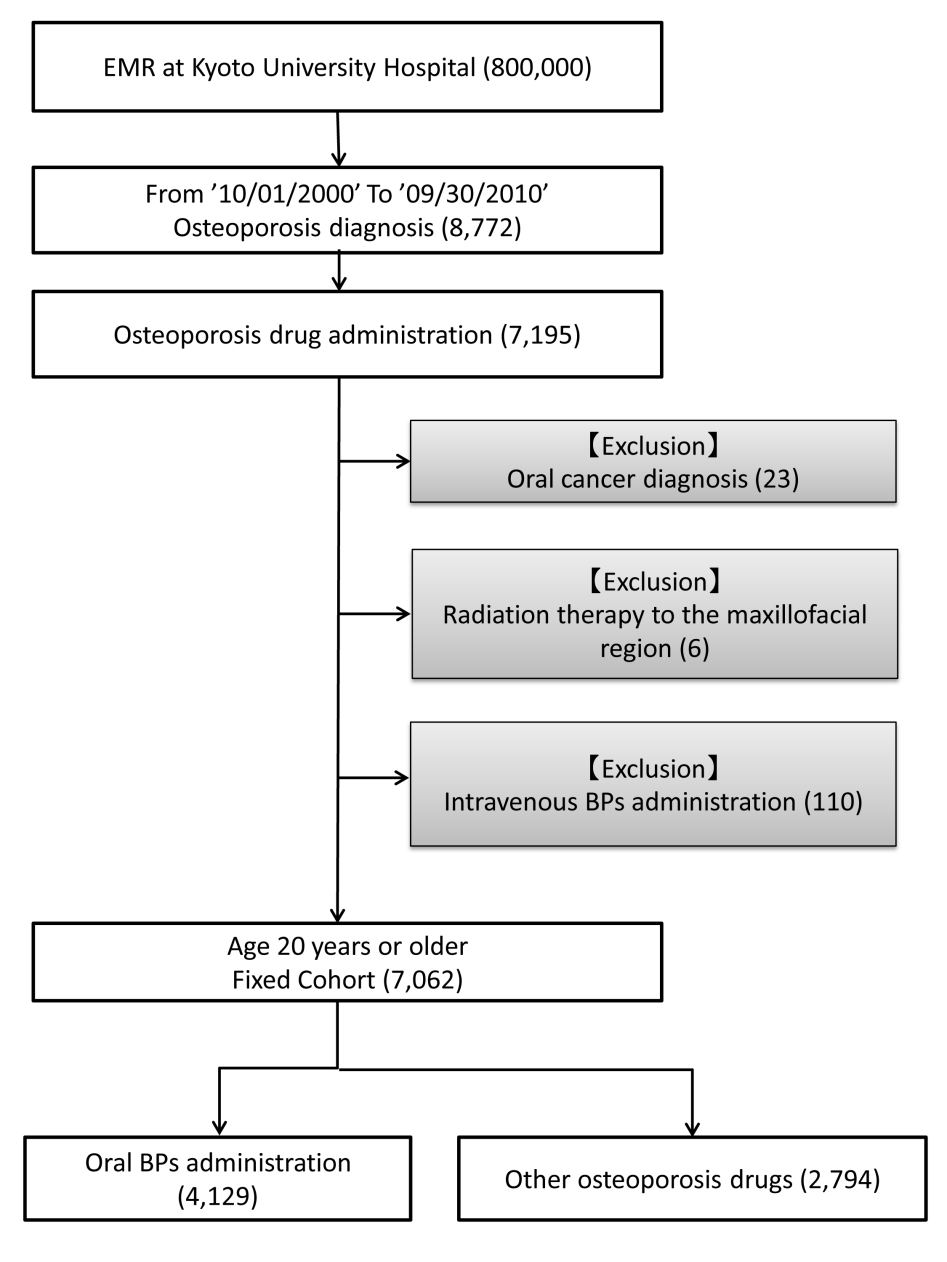
Patient flowchart. BPs = Bisphosphonates; EMR = Electronic Medical Records.

Figure 1 shows a patient flowchart. A total of 7,062 patients treated with osteoporosis medications and aged 20 years or older were included. After exclusion of 29 patients with primary or metastatic tumors in the oral region and/or a history of craniofacial radiation therapy and 110 patients receiving intravenous BPs, 6,923 (98.0%) eligible patients were entered into the analysis.

Patient characteristics are summarized in Table 1. The total number of patients prescribed oral BPs was 4,129 (59.6%), while 2,794 (40.3%) received other osteoporosis drugs. Prevalence of diabetes in our cohort was higher than in the Japanese population [29]. Steroid users accounted for approximately 60% of all patients and 70% in the subset of oral BP users.

**Table 1:** Characteristics of all patients taking any medications for osteoporosis and the subset of oral bisphosphonate users.

	Osteoporosis patients			Bisphosphonate users	
	(n = 6,923)			(n = 4,129)	
Median age (range)	65.0	(20-99)		65.0	(20-99)
Male, *n* (%)	1,539	(22.2)		814	(19.7)
Diabetes, *n* (%)	1,149	(16.6)		707	(17.1)
Steroid use, *n* (%)	4,442	(64.1)		2,934	(71.0)
Chemotherapy use, *n* (%)	807	(11.6)		551	(13.3)
Antirheumatic drugs use, *n* (%)	1,265	(18.2)		977	(23.6)
Biologic agents use, *n* (%)	186	(2.6)		145	(3.5)
Oral BPs administration*					
Etidronate, *n* (%)	N.A.			548	(13.2)
Alendronate, *n* (%)	N.A.			2,871	(69.5)
Risedronate, *n* (%)	N.A.			1,604	(38.8)
Minodronate, *n* (%)	N.A.			38	(0.92)

BPs = Bisphosphonates; N.A. = Not applicable.

* In some cases, several oral BPs were prescribed for one patient.

Table 2 shows potential risk factors for OMJ in all osteoporosis patients and in those limited to oral BP users. Forty-six patients developed OMJ (0.66%, 95% CI: 0.47-0.85) among all patients, and 41 developed OMJ (0.99%, 95% CI: 0.69-1.2) among oral BPs users. In the analysis of all patients, oral BPs were shown to be a strong risk factor for OMJ (OR: 4.98, 95% CI: 1.94-12.75). Age was also a significant risk factor of OMJ (OR: 1.28, 95% CI: 1.03-1.60), whereas sex, diabetes, corticosteroids, cancer chemotherapy, antirheumatic drugs and biologic agents were not associated with the incidence of OMJ. In analysis of the interaction of two potential risk factors pairs, sex-chemotherapy showed an increased risk of OMJ (OR: 11.70, 95% CI: 1.46-93.64). Similarly, in analysis among patients limited to oral BP users, significant associations were seen for age, sex and sex-chemotherapy.

**Table 2:** Potential risk factors of osteomyelitis of the jaw in all osteoporosis patients and oral bisphosphonate users.

	Osteoporosis patients (46 cases, 6,923 patients)						Bisphosphonate users (41 cases, 4,129 patients)				
	Regression coefficient	Odds ratio	95% confidence interval		p		Regression coefficient	Odds ratio	95% confidence interval		p
Oral bisphosphonates, use vs. non-use	1.605	4.98	1.94	12.75	<0.01		-				
Age, +10 years	0.247	1.28	1.03	1.60	0.03		0.269	1.31	1.03	1.67	0.03
Sex, men vs. women	-0.911	0.40	0.14	1.14	0.09		-1.493	0.22	0.05	0.95	0.04
Diabetes, yes vs. no	0.574	1.78	0.86	3.68	0.12		-0.050	0.95	0.41	2.18	0.91
Steroid, use vs. non-use	-0.209	0.81	0.36	1.84	0.62		0.509	1.66	0.78	3.56	0.19
Chemotherapy, use vs. non-use	-0.925	0.40	0.09	1.66	0.21		-0.890	0.41	0.10	1.73	0.22
Antirheumatic drugs, use vs. non-use	-0.045	0.96	0.44	2.08	0.91		-0.294	0.75	0.31	1.77	0.51
Biologic agents, use vs. non-use	0.756	2.13	0.57	7.91	0.26		1.007	2.74	0.71	10.60	0.14
Sex-chemotherapy interaction	2.460	11.70	1.46	93.64	0.02		3.067	21.48	2.14	215.21	0.01

*Risk indices were calculated by the sum of risk factors of an individual patient multiplied by the regression coefficient.

**Figure 2:**
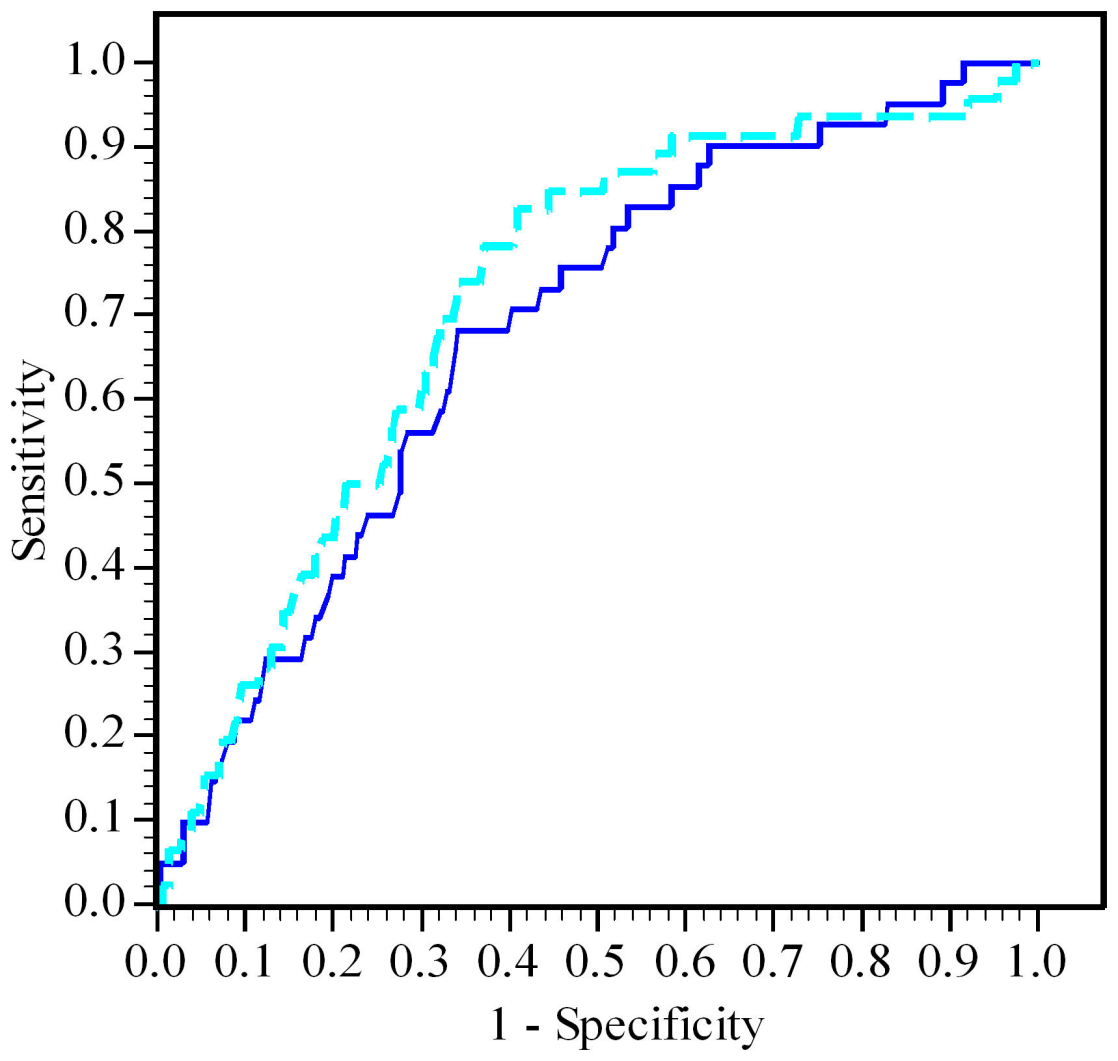
Receiver operating characteristic curves of risk indices in predicting the incidence of OMJ. Areas under the curve are 0.683 (95% confidence interval, 0.607 to 0.760) for the solid curve among oral BPs users and 0.718 (0.648 to 0.789) for the dashed curve in all patients.

Figure 2 shows ROC curves of the risk indices for all patients and oral BPs users only in predicting the incidence of OMJ. Area under the curve was 0.683 (95% CI: 0.607- 0.760) among oral BPs users and 0.718 (95% CI: 0.648-0.789) in all patients. All curves provided only moderate sensitivity or specificity in predicting the incidence of OMJ.

## Discussion

Our study had two major findings. First, we clarified three systemic risk factors for OMJ in patients with osteoporosis, namely age, oral BP use and sex-chemotherapy. In contrast, diabetes, or the use of corticosteroids, antirheumatic drugs, and biologic agents were not identified as significant risk factors of OMJ. Second, our ROC analysis of risk indices of systemic risk factors, to our knowledge the first time this has been investigated, found that ROC curve provided moderate sensitivity or specificity to allow the prediction of OMJ incidence in subsets of patients at high risk of OMJ.

Previous studies did not clarify in detail the association between potential systemic risk factors and the incidence of OMJ in osteoporosis patients nor the accuracy of prediction based on such risk factors [2-10], although they did examine the relative risk of oral BPs after adjustment for these factors. In contrast, while diabetes, corticosteroids use and cancer chemotherapy have all been suggested to be risk factors of OMJ in cancer patients [30-37], diabetes and corticosteroids use were not significant risk factors of OMJ in our cohort. This result appears contradictory. We speculate that differences in the target populations of the studies influenced systemic risk factors of OMJ; in other words, patients with osteoporosis likely received quite different treatment from cancer patients, which in turn resulted in different susceptibility to OMJ. In addition, we suspect that differences in systemic risk factors were partly due to differences among the search procedures used to identify risk factors in the various studies. In particular, almost all these factors were investigated as possible confounding factors or secondary endpoints in the studies, but given that some surveys were conducted using questionnaires, interview, or chart review, and that detailed definitions of most factors were not provided, the accuracy of some diagnoses might have been low. On the contrary, data extraction from the EMR in this study was conducted using an EMR retrieval system [19], and the confirmation of risk factors was defined by rigorous diagnostic criteria [19]. This comprehensive data extraction process and confirmation of risk factors likely improve the reliability of our results.

Our ROC analysis suggests that other risk factors contribute to the incidence of OMJ. As one such predictor, oral bacteria, are hypothesized to confer risk, given that osteomyelitis in BPs users develops only in the jawbones [14], albeit that our present and previous studies have not proved the risk of poor oral hygiene or oral bacteria at the population level. A role for genetic factors has also been hypothesized, but even recent studies of genetic factors lacked sufficient statistical power to predict the incidence of OMJ [38-40]. Further investigations to examine other predictors of the incidence of OMJ in patients with osteoporosis are required.

Nevertheless, our findings do provide relevant information to support decision-making by practitioners involved in the treatment of patients taking oral BPs. At the initiation of BP use, physicians or pharmacists may predict the risk of OMJ incidence according to the clinical characteristics of patients and consult oral specialists in advance. Dentists or oral and maxillofacial surgeons may also consider these risk factors in their care of patients using oral BPs, and the modification of practice patterns is likely useful in preventing OMJ incidence.

Several limitations of the study warrant mention. First, selection bias is inherent to single-center studies, and the present study was additionally subject to inherent referral bias toward the selection of more severe cases, given that our hospital is a lead institution in Kyoto City. Patient characteristics at our hospital might therefore differ somewhat from those at other hospitals or clinics. Second, although our estimation models adjusted for potential risk factors, including age, sex, diabetes, use of oral BPs, corticosteroids, cancer chemotherapy, antirheumatic drugs, and biologic agents and their interactions, no adjustment was made for other possible risk factors related to OMJ, such as smoking or oral BP dose, etc.[41]. However, several studies reported that there was no association between dose of oral BP or other risk factors and OMJ [5,6,9], and risk factors of BPs-related OMJ are controversial. Third, we were unable to examine in detail the primary diseases, severity of illness, and drug dosages in our cohort, although these factors may be associated with risk of OMJ.

In conclusion, our data suggest that oral BP use is strong risk factor for OMJ, and that age and sex-chemotherapy are also systemic risk factors in osteoporosis patients. The risk indices are moderately high, and allow the prediction of OMJ incidence.

## Supporting Information

Appendix S1 **Diagnoses and 10th International Classification of Diseases codes for osteoporosis.**
(DOCX)

Appendix S2 **Drug and generic names for osteoporosis medications approved in Japan between November 2000 and October 2010.**
(DOCX)

Appendix S3 **Diagnoses and 10th International Classification of Diseases codes for case definition for osteomyelitis or osteonecrosis of the Jaw (version 2007, updated in January 2010).**
(DOCX)
