# Supplementary material for: Risk Factors and Indices of Osteomyelitis of the Jaw in Osteoporosis Patients: Results from a Hospital-Based Cohort Study in Japan
Source: PLoS One. 2013 Nov 1;8(11):e79376. doi: 10.1371/journal.pone.0079376 (PMC3815193; doi:10.1371/journal.pone.0079376)
Supplement: Appendix S1 — Diagnoses and 10th International Classification of Diseases codes for osteoporosis. (DOCX) [file pone.0079376.s001.docx]

**Appendix S1** Diagnoses and 10th International Classification of Diseases codes for osteoporosis.

| Diagnosis | ICD-10 code |
| --- | --- |
| **Postmenopausal osteoporosis with pathological fracture** | M80.0 |
| **Postoophorectomy osteoporosis with pathological fracture** | M80.1 |
| **Osteoporosis of disuse with pathological fracture** | M80.2 |
| **Postsurgical malabsorption osteoporosis with pathological fracture** | M80.3 |
| **Drug-induced osteoporosis with pathological fracture** | M80.4 |
| **Idiopathic osteoporosis with pathological fracture** | M80.5 |
| **Other osteoporosis with pathological fracture** | M80.8 |
| **Unspecified osteoporosis with pathological fracture** | M80.9 |
| **Postmenopausal osteoporosis** | M81.0 |
| **Postoophorectomy osteoporosis** | M81.1 |
| **Osteoporosis of disuse** | M81.2 |
| **Postsurgical malabsorption osteoporosis** | M81.3 |
| **Drug-induced osteoporosis** | M81.4 |
| **Idiopathic osteoporosis** | M81.5 |
| **Localized osteoporosis [Lequesne]** | M81.6 |
| **Other osteoporosis** | M81.8 |
| **Osteoporosis, unspecified** | M81.9 |
| **Osteoporosis in multiple myelomatosis** | M82.0 |
| **Osteoporosis in endocrine disorders** | M82.1 |
| **Osteoporosis in other diseases classified elsewhere** | M82.8 |
