# Supplementary material for: Risk Factors and Indices of Osteomyelitis of the Jaw in Osteoporosis Patients: Results from a Hospital-Based Cohort Study in Japan
Source: PLoS One. 2013 Nov 1;8(11):e79376. doi: 10.1371/journal.pone.0079376 (PMC3815193; doi:10.1371/journal.pone.0079376)
Supplement: Appendix S2 — Drug and generic names for osteoporosis medications approved in Japan between November 2000 and October 2010. (DOCX) [file pone.0079376.s002.docx]

**Appendix S2** Drug and generic names for osteoporosis medications approved in Japan between November 2000 and October 2010

| Drug names | Generic names |
| --- | --- |
| Active Vitamin D_3_ Preparation | Alfacalcidol |
|  | Calcitiriol |
|  |  |
| Vitamin K_2_ Preparation for treatment of Osteoporosis | Menatetrenone |
|  |  |
| Bisphosphonates | Alendronate sodium hydrate |
|  | Sodium risedronate hydrate |
|  | Etidronate disodium |
|  | Minodronic acid hydrate |
|  |  |
| Estrogens | Estradiol |
|  | Estriol |
|  |  |
| Selective Estrogen Receptor Modulators | Raloxifene hydrochloride |
|  |  |
| Calcium Supplements | Calcium L-aspartate |
|  | Dibastic calcium phosphate |
|  |  |
| Hormone analogues | Ipriflavone |
|  | Metenolone acetate |
|  | Metenolone enanthate |
|  | Nandrolone cyclohexylpropinate |
|  | Nandrolone decanoate |
|  | Mestanolone |
