# Supplementary material for: Risk Factors and Indices of Osteomyelitis of the Jaw in Osteoporosis Patients: Results from a Hospital-Based Cohort Study in Japan
Source: PLoS One. 2013 Nov 1;8(11):e79376. doi: 10.1371/journal.pone.0079376 (PMC3815193; doi:10.1371/journal.pone.0079376)
Supplement: Appendix S3 — Diagnoses and 10th International Classification of Diseases codes for case definition for osteomyelitis or osteonecrosis of the Jaw (version 2007, updated in January 2010). (DOCX) [file pone.0079376.s003.docx]

**Appendix S3** Diagnoses and 10th International Classification of Diseases codes for case definition for osteomyelitis or osteonecrosis of the Jaw (version 2007, updated in January 2010).

| **First step** | ICD-10 code | Second step | ICD-10 code |
| --- | --- | --- | --- |
| **Inflammatory conditions of jaws** |  | **Benign neoplasm of bone and articular cartilage** | |
| Osteitis **of jaws** | K10.2 | **Bones of skull and face** | D16.4 |
| Osteomyelitis **of jaws** | K10.2 | Maxilla (superior) |  |
| (Neonatal) osteomyelitis **of jaws** | K10.2 | Orbital bone |  |
| Osteoradionecrosis **of jaws** | K10.2 | **Lower jaw bone** | D16.5 |
| Periostitis **of jaws** | K10.2 | **Benign neoplasm of mouth and pharynx** | D10 |
| Sequestrum of jaw bone | K10.2 | **Diseases of pulp and periapical tissues** | K04 |
| **Alveolitis of jaws** |  | **Gingivitis and periodontal diseases** | K05 |
| Alveolar osteitis | K10.3 | **Cysts of oral region, not elsewhere classified** | K09 |
| Dry socket | K10.3 | **Stomatitis and related lesions** | K12 |
| **Disease of jaws, unspecified** | K10.9 | **Cutaneous abscess, furuncle and carbuncle** | |
|  |  | **Face** | L02.0 |
|  |  | **Neck** | L02.1 |
|  |  | Other sites | L02.8 |
|  |  | **Unspecified** | L02.9 |
|  |  | **Cellulitis** |  |
|  |  | **Face** | L03.2 |
|  |  | Other sites | L03.8 |
|  |  | **Unspecified** | L03.9 |
|  |  | Osteomyelitis | M86 |
|  |  | Osteonecrosis | M87 |
|  |  | **Fracture of skull and facial bones** | S02 |
